# MiStImm: an agent-based simulation tool to study the self-nonself discrimination of the adaptive immune response

**DOI:** 10.1186/s12976-019-0105-5

**Published:** 2019-05-02

**Authors:** Csaba Kerepesi, Tibor Bakács, Tamás Szabados

**Affiliations:** 1 Institute for Computer Science and Control, Hungarian Academy of Sciences, Kende u 13-17, Budapest, 1111 Hungary; 2 Alfréd Rényi Institute of Mathematics, Hungarian Academy of Sciences, Reáltanoda u 13-15, Budapest, 1053 Hungary; 3 Department of Stochastics, Budapest University of Technology and Economics, Müegyetem rkp 3, Budapest, 1521 Hungary

**Keywords:** Immune system simulation, Self-nonself discrimination, Self-centered model, Agent-based model

## Abstract

**Background:**

There is an increasing need for complex computational models to perform in silico experiments as an adjunct to in vitro and in vivo experiments in immunology. We introduce Microscopic Stochastic Immune System Simulator (MiStImm), an agent-based simulation tool, that is designed to study the self-nonself discrimination of the adaptive immune system. MiStImm can simulate some components of the humoral adaptive immune response, including T cells, B cells, antibodies, danger signals, interleukins, self cells, foreign antigens, and the interactions among them. The simulation starts after conception and progresses step by step (in time) driven by random simulation events. We also have provided tools to visualize and analyze the output of the simulation program.

**Results:**

As the first application of MiStImm, we simulated two different immune models, and then we compared performances of them in the mean of self-nonself discrimination. The first model is a so-called conventional immune model, and the second model is based on our earlier T-cell model, called “one-signal model”, which is developed to resolve three important paradoxes of immunology. Our new T-cell model postulates that a dynamic steady state coupled system is formed through low-affinity complementary TCR–MHC interactions between T cells and host cells. The new model implies that a significant fraction of the naive polyclonal T cells is recruited into the first line of defense against an infection. Simulation experiments using MiStImm have shown that the computational realization of the new model shows real patterns. For example, the new model develops immune memory and it does not develop autoimmune reaction despite the hypothesized, enhanced TCR–MHC interaction between T cells and self cells. Simulations also demonstrated that our new model gives better results to overcome a critical primary infection answering the paradox “how can a tiny fraction of human genome effectively compete with a vastly larger pool of mutating pathogen DNA?”

**Conclusion:**

The outcomes of our in silico experiments, presented here, are supported by numerous clinical trial observations from the field of immunotherapy. We hope that our results will encourage investigations to make in vitro and in vivo experiments clarifying questions about self-nonself discrimination of the adaptive immune system. We also hope that MiStImm or some concept in it will be useful to other researchers who want to implement or compare other immune models.

**Electronic supplementary material:**

The online version of this article (10.1186/s12976-019-0105-5) contains supplementary material, which is available to authorized users.

## Background

We are witnessing a major change in immunology’s conceptual character from an emphasis on immunity as a defense to immunity as an interface-of-exchange. Immunity should be regarded as a communicative system of the internal homeostasis which perceives and then mediates environmental information (organic and inorganic; internal and external) [1]. To handle this complexity there is an increasing need for complex computational models to perform in silico experiments as an adjunct to in vitro and in vivo experiments. One of the key points of immunity is the concept of self-nonself discrimination. We proposed first that in order to recognize self and non-self, T lymphocytes should recognize the much smaller set of self antigens, rather than the practically unlimited non-self antigen universe [2, 3]. The immune system is continuously in a state of delicate balance between tolerating self and attacking non-self. If this balance is perturbed, autoimmune reactions occur. Immunological tolerance is rooted in regulatory immune cell subsets, suppressive cytokines, and immune checkpoint pathways [4].

A good example for the delicate balance between immune tolerance and intolerance – and for the importance of this research area – is the ambiguous results of The Cancer Immunotherapy Revolution [5], in which the newly approved immunotherapies manipulate components of the immune system to attack tumors. Hundreds of clinical trials are underway to improve responses and success stories of terminal cancer patients defying the odds and achieving complete remissions are accumulating. Unfortunately, the manipulation of the immune system has also resulted in a major safety issue: the iatrogenic immune-related adverse events (IrAEs). As a result of the impaired self-tolerance, irAEs may present with a broad clinical spectrum that mainly involves the gut, skin, endocrine glands, liver, and lung but can potentially affect any tissue, and their incidence may reach up to 90% of patients [6, 7].

In order to aid in the qualitative characterization and examination of the delicate immune balance, we have developed MiStImm computer program, which is capable to simulate the complex processes of self-nonself discrimination of the adaptive immune system. We know that a computer model can not reliably simulate the whole immune system, however, simulating areas of interest can be useful for testing ideas to help in the design of in vivo and in vitro experiments [8].

MiStImm uses agent-based modeling technique [9] and it can simulate some aspects of humoral immune response along with its major components, including T cells, B cells, antibodies, danger signals, interleukins, self cells and foreign antigens. These simulation components (called “agents”) determine the nodes of a dynamic immune network where links are the potential interactions between two elements. The immune network changes step by step (in time) driven by random events. Using the terminology of [10], a model simulated by MiStimm is an agent-based model that is in part “individual particle based-stochastic”, and in part “particle number stochastic”. An “individual particle based stochastic element” is an agent that models individual cells and their individual random attachments with other cells or molecules. In our program, this approach is used for Th cells and B cells. A “particle number stochastic element” is a population of cells or molecules that are represented in the model by the properties of the population and by the number of elements in it. In our program, this approach is used e.g. for self cells and foreign antigens. Because our model is stochastic, their attachments with other elements is also controlled randomly. A great advantage of such a model is that it can easily incorporate the most important types of cells and molecules together with their essential features and simulation events that play important roles in immune reactions. In such a simulation events – for example interactions of components – occur at random. A stochastic model fits well with the affinity maturation of B lymphocytes in which random events are perhaps the most characteristic. It is also suitable to model the development of the regulatory T cell population and the random selection of specific T cell clones.

To simplify things, we chose the humoral adaptive immune system since the humoral phase (blood or lymph) may be considered spatially homogeneous; thus a microscopic spatial volume may represent the whole phase well. A major advantage of this approach is that it is not necessary to describe the actual spatial positions and spatial motions in the model. Instead, model components (agents) randomly choose one of the other components as interaction partners, because any components are close enough to become engaged in an interaction.

As the first application of MiStImm, we have simulated two different immune models and then we have compared performances of them in the mean efficacy of self-nonself discrimination (see the Results). The first model is called *nonself centered* or *Conventional Role of Self* (CRS) where even a primary immune reaction depends on the recognition of non-self antigens by T and B cell receptors [11–13]. The role of self in this model is that the great majority of autoreactive T and B cell clones are selected and purged from the immune system [14]. The second model called *self-centered* or *Enchanced Role of Self* (ERS) which is based on our previously published “one-signal model” [3]. We proposed that model (hypothesis) when we have been seeking the answer to three unresolved paradoxes of immunology:

- (Q1) How can a tiny fraction of human genome effectively compete with a vastly larger pool of mutating pathogen DNA [15]?
- (Q2) Considering the fact, that average 3 mutations are formed each of the 10^16^ times the cell’s 3·10^9^ DNA base pairs are duplicated during a human lifetime [16], “why does cancer occur so infrequently”?
- (Q3) Considering the facts that T cells require three to five days to attain fighting strength (because they are rare, short-lived, and their doubling time is at least 6 h), yet how can a T cell response be measurable in the lymph nodes draining the infection site within 12 to 18 h [17]?

**Fig. 1:**
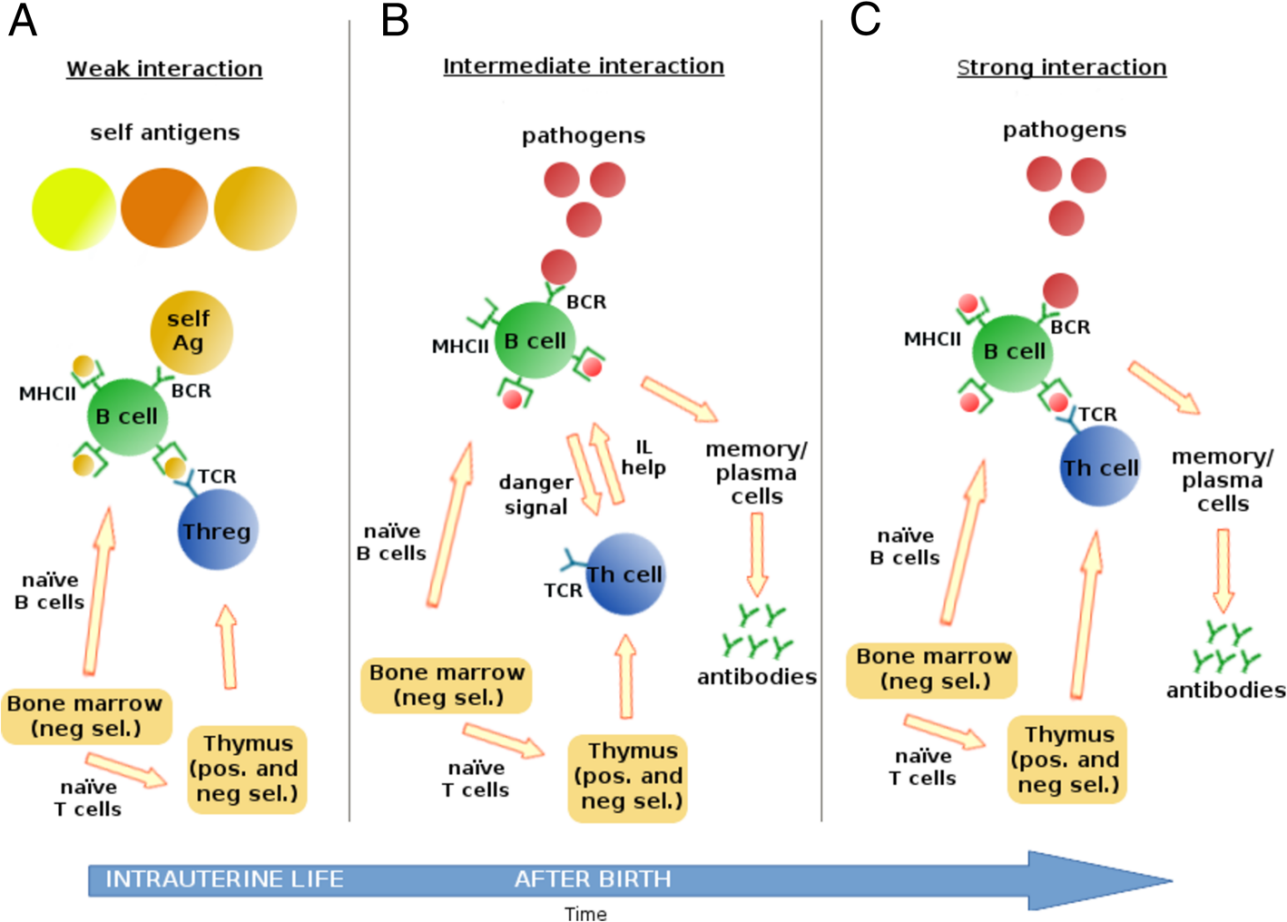
Humoral adaptive immune response by the ERS and CRS model. The ERS model is described by (**a**), (**b**) and (**c**), while CRS models are described by (**c**) alone. **a** In the ERS model, a hypothesized *weak affinity interaction* begins in intrauterine life and keeps the immune image of self during the whole life. It is sufficient for homeostasis; low affinity BCR binds self-antigens and presents self-peptides in their MHCII to regulatory T helper (Threg) cells; this ensures B and Threg cell survival. **b** In the ERS model another hypothesized interaction, *intermediate affinity interaction* initiate the first line of defense against an infection; some B cells that have higher BCR affinity for the antigens of the pathogen capture pathogens with intermediate affinity and present foreign peptides in their MHCII. The foreign peptides indirectly inhibit binding of Threg cells to these B cells for a critical time period, then the B cells will secrete hypothesized danger signals. Danger signals activate local Th cells, which in turn, release interleukins that fuel local T cell activation. This way a non-specific, local polyclonal B and T cell activation is induced, which is the primary defense mechanism against infections in the ERS model. Clonal expansion requires affinity maturation, which results in a several magnitude increase of BCR affinity, typically over a time of one week. Random mutations cause the production of B cells with a broad range of affinities for their presented foreign antigen. B cells with unfavorable mutations will not get sufficiently activated by the foreign antigen and will die, while those with improved affinity will be stimulated to clone themselves. **c** Specific immune reaction, here called as *strong affinity interaction*, appears in both the ERS and CRS models and is supervised and supported by pathogen peptide-specific Th cells, which require direct contact via TCR to the MHCII of the expanding B cell clone. Such higher affinity interactions would then drive clonal T cell proliferation, activation, lysis of infected cells. Having cleared the infection, specific T cells could eventually become an expanded memory type T cell clone, while B cells could differentiate into infection specific antibody-producing plasma cells or memory B cells. This interaction usually needs several days to efficiently start

In order to explain these paradoxes, we have suggested a new T cell model [3] that we can summarize in the following. We have postulated that a dynamic steady state, a so-called coupled system is formed through low affinity complementary TCR–MHC interactions between T cells and host cells. Under such condition, it is sufficient to recognize what is self in order to attack nonself (answer to Q1). We have postulated that the evolutionary pressure driving the creation of the T cell receptor (TCR) repertoire was primarily the homeostatic surveillance of the genome (answer to Q2). The new model implies that a significant fraction of the naive polyclonal T cells is recruited into the first line of defense from the very outset of an infection (answer to Q3). The computational variant of our hypothesized T-cell model is the ERS model, presented in this paper. The ERS and CRS model are summarized by Fig. 1.

Though, there are some immune system simulation models that are capable to simulate a conventional (or standard) immune model like our CRS model (e.g. Basic Immune Simulator [18], C-ImmSim [19, 20], SIMISYS [21]), they are not directly usable for modelling the self-nonself discrimination theory of our group (presented by the ERS model) and compare it with a conventional model. However, when we built MiStImm, we have adapted some principles of the earlier simulation models (see the Discussion for a comparison).

The main goal of the simulation experiments of the current paper is showing that the ERS model matches real patterns and additionally to analyze how the two models (CRS and ERS) cope against a critical primary infection. That was the main reason why we have developed MiStImm.

In the Results we show that the ERS model does not develop autoimmune reactions despite the existence of the hypothesized TCR–MHC interaction between T cells and self antigens in the model. Autoimmune reaction is a strong immune response of an organism against its own healthy cells and tissues. Despite the weak reaction of B and Th cells against the healthy self cells in the model, the sizes of these self cell populations are not decreasing, so does not occur a pathological consequence of these weak reactions.

We also show that the ERS model gives better results to overcome a critical primary infection, answering the paradox “how can a tiny fraction of human genome effectively compete with a vastly larger pool of mutating pathogen DNA?” We hope that our results will encourage investigations to make in vitro and in vivo experiments clarifying questions about self-nonself discrimination of the adaptive immune system. We also hope that MiStImm or some concept in it will be useful for implementation and/or comparing other immune models by other researchers.

## Implementation

The Microscopic Stochastic Immune Simulator (MiStImm) is a single program written in the C programming language in the spirit of agent-based models [9]. We have provided its executed binary file for instant use in Windows operation system. It gets its input parameters from a raw text file. By changing the input parameters new initial conditions or even new immune models can be set up for the simulation program (see the list of parameters in Additional file 1). The output of the simulation consists of two text files containing the state of the system at all time steps with additional statistics. We have also provided additional tools to visualize and analyze the output of the simulation program.

### Components (agents) of the simulation

MiStImm can simulate some components of the humoral adaptive immune system, like helper T cells, B cells, antibodies, interleukins, self cells, and foreign antigens (Table 1). Components (agents) are handled as population or individuals and each of them is implemented as a custom data type in C (called “struct” that is similar to a “class” in other computer languages like C++). Each component has a number of numerical parameters and certain attached events or processes of events that may occur at random (see details in Table 1). The output of the simulator contains the number of each components at each time point so we can trace the changes of the population sizes over a simulation (see simulation outcomes in the Results). In nature, it is typical that when the size of a certain cell population gets larger the per capita birth rate in the population decreases. Thus the size of a population first increases fast, later it slows down, and at the end it gets relatively stable. So to control birth rates (ensuring that the number of components always remain in the biologically feasible domain) we have used logistic functions previously applied by many other authors (see e.g. [22, 23] and Additional file 1).

**Table 1:** Associated events and behaviors of the simulated components (“agent types”) in MiStImm program

Agent type	Simulation event or behavior	Model
Self cell pop.	Born	ERS/CRS
Foreign antigen pop.	Born	ERS/CRS
Bone marrow cell pop.	Born	ERS/CRS
	Creates a native B cell	ERS/CRS
	Creates a T helper cell	ERS/CRS
B cell (individual)	Born at the periphery by division	ERS/CRS
	Negative selection in the bone marrow	ERS/CRS
	Dies because its lifespan terminates	ERS/CRS
	Emits some danger signals	ERS
	Activation control process	ERS
	Action*:	ERS/CRS
	- detection/killing/antigen presentation	ERS/CRS
	- division of weak kind	ERS
	- division of intermediate kind	ERS
	- division of strong kind	ERS/CRS
	A plasma cell creates some antibodies	ERS/CRS
Th cell (individual)	Born at the periphery	ERS/CRS
	Dies because its lifespan terminates	ERS/CRS
	Positive and negative selection in the thymus	ERS/CRS
	Emits some interleukin	ERS
	Activation control process	ERS
	Action*:	ERS/CRS
	- detection	ERS/CRS
	- division of weak kind	ERS
	- division of intermediate kind	ERS
	- division of strong kind	ERS/CRS
Danger signal pop.	Some danger signals die	ERS
	Action*	ERS
Interleukin pop.	Some interleukins die	ERS
	Action*	ERS
Antibody pop.	An amount of antibodies dies	ERS/CRS
	An amount of antibodies acts (kill)*	ERS/CRS

B and Th cells are handled individually while the remaining types of agents are handled as population (“pop”.). Each row in the table describes a scheduled event of the simulation except the subprocesses of the *Action events*. These subprocesses can occur along an *Action* event. Interactions among agents can also occur as an implication of the five action events (signed with an asterisk*). Some simulation events and subprocess are elements of both the ERS and the CRS model, others are elements only the ERS model. A plasma cell is a special kind of B cells, a result of a B cell maturity process. A plasma cell has neither a B cell action event nor a B cell activation control event. On the other hand, it has an antibody birth and an antibody death event. An antibody has the same shape in the antigen lattice as the BCR of its mother plasma cell

### Progression of simulation events

A simulation progresses with discrete time points by consecutive steps (turns). At each time point a simulation *event* is selected randomly from the actual list of the scheduled events (see possible events in Table 1). Then the selected event occurs and it changes the state of the system: it can change the state of the simulation components (agents) and also can include/remove/modify other scheduled events in the list. The random selection (and the time point of the occurrence) depends on the expected waiting times of the events in the actual scheduled event list. (See details in the “Mathematical model” section of the Additional file 1). In the program, each event is implemented as a C function containing the rules that will be executed when the event occurs (see the details of the rules below and in Additional file 1). The scheduled simulation event list is programmed as a dynamic list of individual objects, consisting of the event ID along with its own parameters like the expected waiting time of the event.

### Simulation spaces

The simulation environment of MiStImm is the spatially homogeneous humoral phase (blood or lymph) of the adaptive immune system. A major advantage of this approach is that it is not necessary to describe the actual spatial positions (locations) and spatial motions in the model. Instead, model components (agents) randomly choose an interaction partner among the other components (agents), because any components are close enough to become engaged in an interaction. Instead of locations, MiStImm simulates the spatial shape of the peptides and receptors that are key points of the adaptive immune response.

#### Simulation space 1: peptide universe

**Fig. 2:**
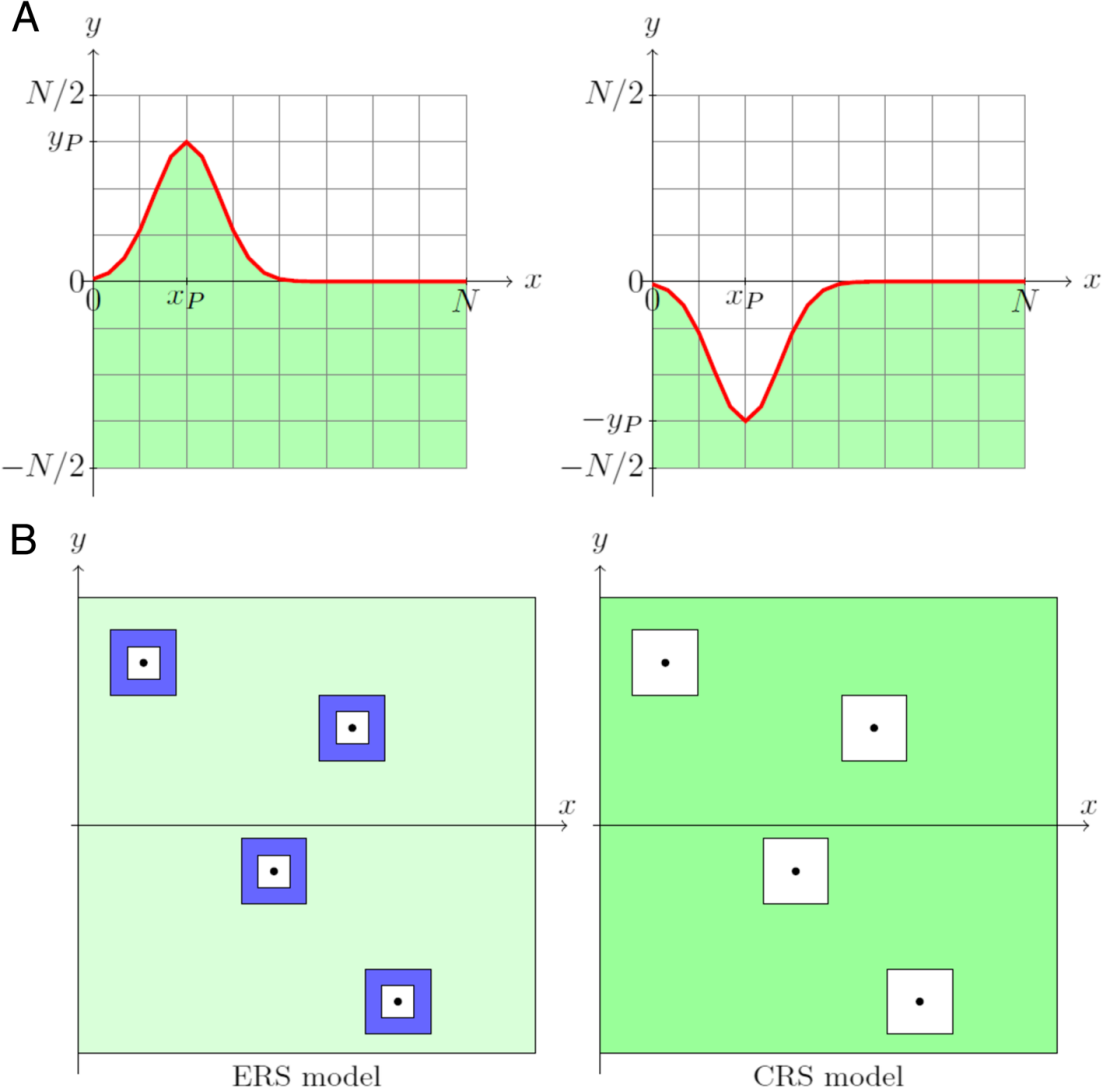
**a** Two simplified complementary shapes characterized by the points (*x*_*P*_,*y*_*P*_) and (*x*_*P*_,−*y*_*P*_), respectively, in the peptide lattice. **b** Simplified graphical representation of the difference between the ERS and the CRS models. Dark blue: area allocated to regulatory T cells; Green: area for potential infection specific T cells; Intensity of the colors represent the density of T cells in the area (a darker color means larger density)

MiStImm takes a microscopic volume of the humoral phase and also a microscopically small part of the shape space universe. Shape space models were used by Perelson, Segel and their colleagues since the 1970’s [24, 25] and also in the Celada–Seiden model [26]. To explain what we mean by shape space here, assume that the shape of a T cell receptor (TCR) can be represented by a point in a discrete lattice of real numbers. Theoretical considerations compared with experimental data led to the conclusion that the dimension of this shape space, i.e. the number of parameters essential in describing a binding, is not too large, probably around five [24]. The microscopically small part of the shape space that we consider in the simulation program is a small discrete *N*×*N* planar grid in the shape space (default: *N*=1000). The *x*∈{0,1,…,*N*} coordinate of a shape point may represent a “horizontal” coordinate of the main part of the binding profile of a TCR or an MHC+peptide complex, while the *y*∈{−*N*/2,…,*N*/2} coordinate may represent the “vertical” coordinate of the main part of the binding profile. A positive coordinate represents “convexity”, while a negative coordinate represents “concavity”. Figure 2a shows our underlying idea for the shape of a peptide characterized by a single point (*x*_*P*_,*y*_*P*_). We call the above finite square grid the peptide lattice.

#### Simulation space 2: antigen universe

The shape of a B cell receptor (BCR) or shape of an antigen is similarly represented by a point of an antigen lattice in the model. Here again the *x*∈{0,1,…,*N*} coordinate of a shape point may represent a “horizontal” coordinate of the main part of the binding profile of the BCR or antigen, while the *y*∈{−*N*/2,…,*N*/2} coordinate may represent the “vertical” coordinate of the main part of the binding profile; a positive coordinate representing “convexity”, while a negative coordinate representing “concavity”, see Fig. 2a.

### Interactions of agents in simulation

#### Complementarity of binding

Complementarity plays a basic role in binding. The perfect fit between a TCR and an MHC+peptide complex means in the model that the shape (*x*_*T*_,*y*_*T*_) of the TCR and the shape (*x*_*P*_,*y*_*P*_) of the MHC+peptide satisfy the equalities *x*_*T*_=*x*_*P*_ and *y*_*T*_=−*y*_*P*_, see Fig. 2a. In the model we introduce *a metric* or *distanced* to measure the degree of similarity of two shapes *z*_1_:=(*x*_1_,*y*_1_) and *z*_2_:=(*x*_2_,*y*_2_):

1 $$ d(z_{1}, z_{2}) := \max \{|x_{2} - x_{1}|, |y_{2} - y_{1}| \}.  $$

(This is called the maximum or *ℓ*_*∞*_ distance in mathematics.) A TCR *z*_*T*_:=(*x*_*T*_,*y*_*T*_) and an MHC+peptide *z*_*P*_:=(*x*_*P*_,*y*_*P*_) are perfectly complementary in our model if the distance between *z*_*T*_ and $\overline {z_{P}} := (x_{P}, -y_{P})$ is zero. The larger the distance, the more imperfect the complementarity is. The representation of the complementarity between BCRs and antigens is similar. Only complementary or nearly complementary shaped ligands and receptors can bind. The dots in Fig. 2b represent TCRs that are exactly complementary to some self MHC+self-peptide complex. In the case of ERS model, the areas shaded in dark blue are called the *characteristic rings of self-peptides*. They represent the set of shapes that are allocated to possible *regulatory T cells* after negative and positive selection in the ERS model. The areas denoted by green correspond to possible shapes of classical, *potentially infection (or mutation) specific T cells*, while white areas are representing self-reactive T cells that are prohibited for T cells in the two respective models. In the ERS model, moderately self-reactive T cells are present after negative and positive selection. In fact, they constitute the most important class of T cells that decide self-nonself discrimination. On the other hand, such moderately self-reactive T cells are negatively selected out in CRS models.

Interactions among agents are implications of the following simulation events: T cell action, B cell action, danger signal action, interleukin action, antibody action (see events signed by asterisks in Table 1). These interactions are realized as BCR – antigen binding, TCR – MHC+peptide binding, or danger signals/interleukins emission (for details see the “Complementarity of binding” section above and Additional file 1). In MiStImm, T cell – B cell interactions are basic, here we describe three different types of it. Each of the three types fulfills an important role in the ERS model (Fig. 1). CRS model can be described by the third type of interactions (called *strong interaction and division*) alone.

#### Weak affinity interaction and division (specific to ERS model)

According to the hypotheses of ERS model, in a healthy individual during intrauterine life, randomly produced moderately self-reactive B cell clones are confronted with an overwhelming quantity of soluble self antigens. Those B cells that can attach with intermediate affinity to any of these self antigens via their B cell receptors (BCRs) will present self peptides in their surface major histocompatibility complex II (MHCII) molecules to regulatory T helper cells (Thregs). This ensures B cell and Threg cell survival, respectively, but it is insufficient to trigger extensive clonally based B cell expansion required for specific immunity or autoimmunity. This hypothesized interaction is called *weak affinity interaction and division* here. Thus the positively selected Threg cells are critical parts of the homeostatic control in our model so that Threg clones exist for practically all kinds of self-MHCII – self-peptide complexes presented by any of the B cells. After birth, this process maintains an immune image of soluble self which can control self-nonself discrimination. This self-surveillance makes ERS model self-centered (rather than infection centered) and gives the answer to the Q2 paradox mentioned in the “Introduction”.

#### Intermediate affinity interaction and division (specific to ERS model)

During a *primary infection* a new antigen appears in the blood. B cells with an appropriate affinity for the new antigen, engulf new antigens and present its foreign peptides on their surface MHCII proteins. Since in the ERS model foreign peptides temporarily inhibit the complementary TCR-MHC interactions, such perturbation creates steric hindrance that obstructs the docking of positively selected Thregs. Disruption of such contact between an MHCII and Thregs *for a critical period of time* results in an emergency and activates the corresponding B cell. In order to reestablish contact, in the ERS model, foreign peptide presenting B cells will secrete a hypothetic *chemotactic danger signals(“smoking gun”)* attracting Th cells to this region. We imagine this process as the B7-1 and B7-2 ligands of B cells will activate most CD28 receptors of the bystander helper T cells, This initiates a non-specific, polyclonal activation in local Th lymphocytes via the CD28 receptor alone [27] such that a local cytokine storm is generated in Th cells triggering B cells to clonal expansion, hypermutation, and eventually they may develop into specific antibody-producing plasma cells. This hypothesized interaction will be called *intermediate affinity interaction and division* here. The resulting inner state of the affected Th and B cells will be called *“activated”* state. Since affinity maturation is driven by the fast increasing local concentration of pathogen antigens (e.g. hepatitis virus), the probability of clonal autoimmunity is very low but possible.

#### Strong affinity interaction and division (specific to both ERS and CRS model)

Both in ERS and CRS model, following the initial polyclonal activation phase, there is always a possibility that rare T cell and B cell clones with higher affinity may well recognize foreign antigens, particularly when a significant fraction of host cells are infected and the viral load is high (for example in hepatitis, see in [28]). Such higher affinity interactions would then drive clonal (e.g. HCV specific) T cell proliferation, activation, lysis of infected cells. Having cleared the infection, specific T cells could eventually become an expanded memory type T cell clone, while B cells could differentiate into infection specific antibody-producing plasma cells or memory B cells. Specific T and B cell activation, proliferation and lysis of infected cells, therefore, obey the rules of the conventional two-signal model. Clearly, this process may require several days in general. This interaction will be called *strong interaction and division* in the sequel. The resulting inner state of the affected Th and B cells is called *“strongly activated”* state here.

#### Hypothesized danger signals and interleukins (specific to ERS model)

We use the symbolic names “danger signals and interleukins” in this paper, without specifying the exact type of these molecules, similarly to Fig. 3 of Ref. [29]. These types of soluble molecules have roles only in intermediate interactions and divisions in the ERS model. Since conventional immune reactions correspond to the ones that we call strong interactions and divisions, these types of molecules do not appear when simulating CRS models. *Danger signals (soluble molecules)* are emitted by B lymphocytes following disruption of the homeostatic complementary interaction of B cells and Threg cells. This event initiates an action event and also a death event of these molecules. Each danger molecule randomly chooses a Th cell agent. This is a signal for the Th cell to start intermediate type division and to secrete interleukins. Note that this danger signal is not the same as in [30] because our danger signals are emitted when the system detects any kind of nonself and not only a dangerous one. *Interleukins* are emitted by Th lymphocytes. This event initiates an action event and also a death event of these interleukins. Each interleukin molecule randomly chooses a B cell. This is a signal for a B cell that has lost complementary Threg cell control to start cell division of intermediate kind. For more details of interactions see in the Additional file 1.

### Parameter setting, model validation

MiStImm can be initialized by 178 of parameters (see Additional file 1). The simulation program, along with its parameter values, was developed by trial and error comparing the simulation outcome patterns to the normal behavior observed in living systems. For example, we set the parameters so that the number of T cells and the number of B cells be approximately equal during a simulation, or we have used logistic functions to prevent population explosion of the components (see details in the Additional file 1). We also used some principles that were tested by other researchers in their work (see Discussion). Simulation outcomes demonstrating our endeavors are shown in the Results.

### Limitations and future perspectives

MiStImm can be easily customized by varying parameters. However, model that is remarkable different from the default settings of MiStImm could be difficult to implement because it requires understanding of the source code written in C. For example, it is easy to vary the type and initial number of foreign and self cells (and many another input parameters), but it is relatively hard to implement a new type of cells (like macrophages) or change the binding model. To solve this limitation an implementation of a Python library would be beneficial providing an easy-to-use interface to customize an immune model from built-in building blocks (similarly as Keras library makes deep learning as easy as manipulating LEGO bricks [31]). We think such a big enterprise could revolutionize immune system modelling. MiStImm is one of the first steps in that road.

## Results

### Simulations of the ERS model show real patterns

Beside providing screenshots of MiStImm simulation tool, the following simulation results demonstrate that the ERS model – introduced above – show real patterns. For example, the ERS model does not develop autoimmune reactions despite the hypothesized enhanced TCR–MHC interaction between T cells and self antigens. We also perform in silico experiments to show the existence of immune memory in the ERS model, and we also show an example of autoimmune reaction at given pathological conditions.

### Simulation of the development and homeostasis of the adaptive immune system by the ERS model

**Fig. 3:**
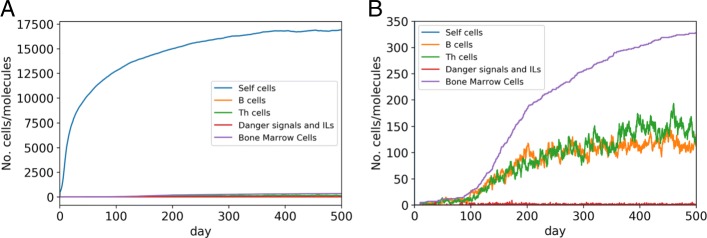
Simulation of the development and homeostasis of the adaptive immune system by the ERS model. The same single simulation in a bird’s eye view (**a**) and a closer view (**b**), respectively. Horizontal axis: time (day) from conception. Vertical axis: number of cells/molecules. In the case of self cells the sum of sizes of the self cell populations is displayed

We have run simulation experiments for the analysis of the development and homeostasis of the simulated immune system by the ERS model. A simulation starts a few days after conception and goes until the 5000th time step; the unit of time is being a tenth of a day (2 h and 24 min). Initially, only three types of non-immune self antigen populations appear in the model, each with a number of 150 cells, and no other components. Each of these populations is accompanied by a cell division process that implies continuous growing of the number of self cells, with decreasing rate in time (Fig. 3a). B and T cells, which generated by the bone marrow cells, first appear at the 10th day (Fig. 3b). The number of these cells also grow continuously at a decreasing rate. According to the ERS model, the immune system does not attack self cells strongly, just to a very limited extent. Some B cells must continuously present self peptides to ensure that Threg cell characteristic rings around self peptides are constantly maintained. Because of negative selection, this type of immune response is weak and typically settles down quickly before it becomes pathological.

**Fig. 4:**
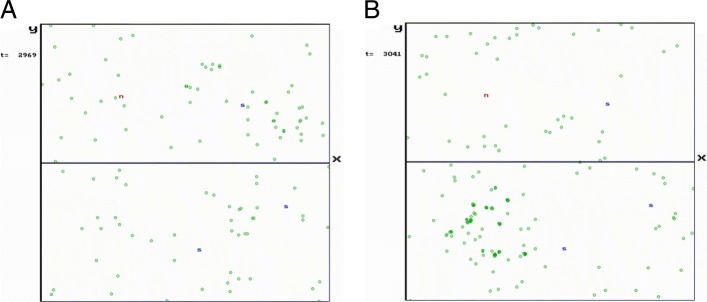
Simulation spaces (peptide and antigen lattices) in the ERS model. **a** A snapshot of the peptide lattice, where the actual TCRs are displayed. With random ‘rings’ around the reflected images of non-immune self antigens (“s”) about one month after birth. A movie capturing a typical simulation of the peptide space is available at the address https://goo.gl/QcdG48. **b** A snapshot of the antigen lattice, where the actually existing BCRs are displayed. B cell response to a pathogen: large density of pathogen specific B cells at the reflected image of nonself (“n”) about one week after the infection. As a result of negative selection, there are empty domains around the reflected images of non-immune self antigens (‘s’). A movie capturing a typical simulation of the antigen lattice (shape space) is also available at the address https://goo.gl/3oK1bM

The peptide and the antigen lattices both have has a size {0,1000}×{−500,500}. Coordinates of antigens of three different self cells (denoted by letters “s”) were (550,300), (700,−200) and (850,150), both in the case of the peptide and the antigen lattice (Fig. 4a and b). TCR rings around the mirror images of self peptides – that are characteristic features of the ERS model – begin to develop about the 150th day and become more or less stabilized by the 280th day (Fig. 4a). These rings fluctuate for two reasons: *(i)* occasionally global Th cell populations overgrow the set upper limits and this reduces the probability of Th cell division; *(ii)* sometimes the number of presented self peptides in the MHCII-peptide complexes of B cells reaches an extremely low level.

### Simulations of the normal immune response and immune memory by ERS model

We have run simulations with the same parameters as above in the development and homeostasis section with the exception that we have injected pathogens into the system at two distinct time points. An infection brings significant changes. A rising population of B and Th cells appear at the mirror image of the infecting agent denoted by a letter “n” (Fig. 4b). An immune response should have the ability to destroy the majority of pathogens – some of them suddenly, others perhaps slowly, while in some cases it may fail. In the ERS model, the death of an individual occurs when the pathogen population grows up irreversibly, technically, as its size reaches 4000 cells. Diversity of pathogens are represented by different locations of their receptors, different speeds of growth, and different initial numbers.

**Fig. 5:**
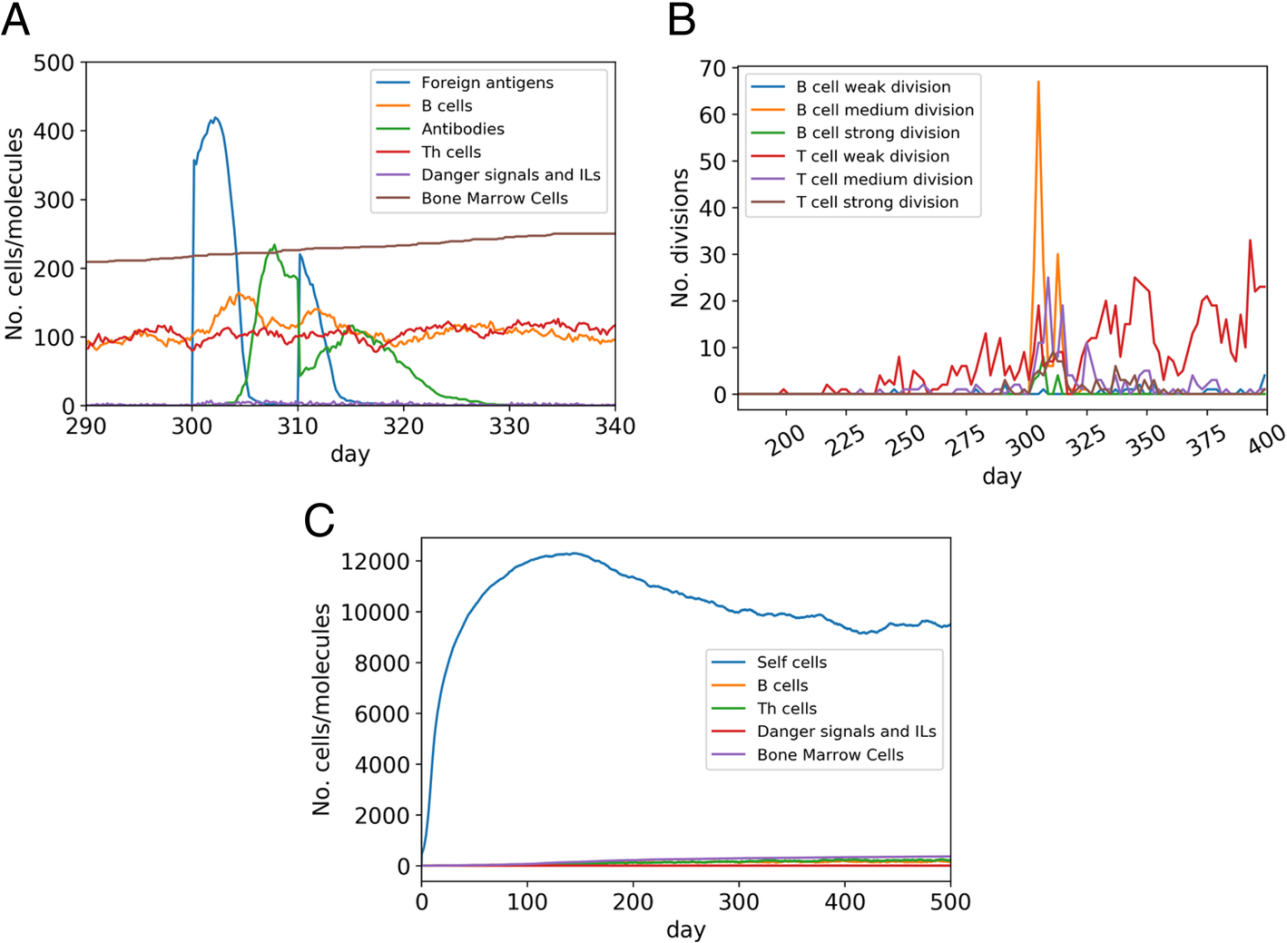
Immune response in the ERS model. **a** Normal immune response against a repeated infection. First infection was injected at the 300th day and the second infection was injected at the 310th day. Both infections are the same type (number of cells =350, mean waiting time between two divisions =60). The second infection was eliminated faster due to the existence of B cell memory. Horizontal axis: time (day) from conception. Vertical axis: number of cells/molecules. **b** Division of weak/intermediate/strong kind of T and B cells in the same simulation showed in (**a**). Horizontal axis: time (day) from conception. Vertical axis: number of divisions. **c** Autoimmunity caused by the lack of negative selection of B cells: number of self cells decreases rapidly. Horizontal axis: time (day) from conception. Vertical axis: number of cells/molecules

A normal immune response develops immune memory. Thanks to memory cells, a second immune response against the same nonself antigen have been more effective than at a primary infection (Fig. 5a and b). For a deeper analysis of the immune memory, we have performed 500 simulations (by the ERS model), adding the same type of pathogen (number of cells =350, mean waiting time between two divisions =60) at the 300th and at the 315th day. ERS model cleared both infections in 451 cases and the mean time lengths needed for elimination were 62.02 (std 13.26) at the first infection and 20.51 (std 14.94) at the second infection. We have said that an elimination happened when the number of pathogens has decreased under 50. With a two-tailed t-test, the *p*-value for equality of mean elimination times for the first and the second infection was 5.2*e*−227.

### Simulation experiment with lack of negative selection of B cells in the ERS model

We want to demonstrate that our simulation tool is capable to simulate autoimmune events at given pathological conditions, for example, the lack of negative selection of B cells is shown in Fig. 5c. Without negative selection some of the B cells can constantly destroy self cells.

### Simulation experiments for comparison of ERS and CRS model against a critical infection

One can switch the ERS (*Enhanced Role of Self*) model to a CRS (*Conventional Role of Self*) model by modifying four parameters. Turning off the *division of weak type* and the *division of intermediate type* are required in the CRS model (medrepr =1→0 and weakrepr =1→0). Turning off the positive selection of T cells is also required in the CRS model (comptype =0→1). The latter adjustment causes large growth of the T cell population, so simultaneously we need to decrease the expectation of the waiting time between two births of T helper cells in the bone marrow (tauthm =5→30). We compared the efficiencies of the immune reactions in the two models. Our results showed that in the ERS model the adaptive immune reaction was able to destroy infections with critically large initial numbers or with critically fast division times more often than in a CRS model (Tables 2 and 3). Fisher’s exact test was used for the statistical evaluation (Table 4). These results show that our ERS model is a proper answer to the paradox Q1, mentioned the Introduction, saying “How can a tiny fraction of human genome effectively compete with a vastly larger pool of mutating pathogen DNA”.

**Table 2:** ERS vs. CRS model, simulated by MiStImm 500–500 times as the initial number of pathogens is increasing (from 200 to 400) and the mean waiting time between two divisions of a pathogen is fixed (50)

f cells	div time	ERS wins	CRS wins	ratio	*p*-value
200	50	499	432	1.155	1.1E-20
250	50	497	361	1.377	1.85E-42
300	50	481	310	1.552	5.19E-45
350	50	417	225	1.853	4.48E-38
400	50	272	135	2.015	5.74E-19

The table shows how the performance of ERS and CRS model is changing when we increase the initial number of pathogens. The unit of time is one-tenth of a day; *f cells*: the initial number of foreign cells at the 300th day; *div time*: the mean waiting time between two divisions of a foreign cell; *ERS wins*: number of wins of the immune system against pathogens using the ERS model setting; *CRS wins*: number of wins of the immune system against pathogens using the CRS model setting; *ratio*: ERS wins divided per CRS wins; *p*-value: one-sided *p*-value of the Fisher’s exact test. In every case ERS performed significantly better than CRS

**Table 3:** ERS vs. CRS model, simulated by MiStImm 500–500 times as the mean waiting time between two divisions of a pathogen is increasing (from 40 to 80) and the initial number of pathogens is fixed (350)

div time	f cells	ERS wins	CRS wins	ratio	*p*-value
40	350	208	66	3.152	1.19E-24
50	350	417	225	1.853	4.48E-38
60	350	473	320	1.478	1.16E-35
70	350	493	400	1.233	1.42E-24
80	350	500	441	1.134	2.81E-19

The table shows how the performance of ERS and CRS model is changing when we increase the mean waiting time between two divisions of a pathogen. Column labels are the same as in Table 2 but the positions of the columns “f cells” and “div time” are switched. In every cases ERS performed significantly better than CRS

**Table 4:** Contingency table of the one sided Fisher’s exact test [54] for the fourth row of Table 2, as an example

	ERS	CRS	Row total
Win	417	225	642
Loss	83	275	358
Col total	500	500	1000

The *p*-value appearing there was calculated by the formula $\sum _{i=417}^{500} {642 \choose i} {358 \choose 500-i} / {1000 \choose 500} \approx 4.48E-38 $. Note that the values of the hypergeometric distribution inside the sum are the probabilities of choosing 500 experiments out of 1000, containing exactly *i* ERS wins of the given 642 total number of wins and also choosing exactly 500−*i* ERS losses of the given 358 total number of losses

### Sensitivity analysis of the model parameters

The objective of a sensitivity analysis (SA) is to identify critical input parameters of a model and quantifying how input uncertainty impacts model outputs [32]. In the previous section, we investigated how a given output (win or lose against a critical infection) changes when we vary one particular input parameter. In this section, we investigate how output variables change when we vary more than one input parameters randomly at the same time. This method gives a more global insight into the correlations between the input and output.

**Fig. 6:**
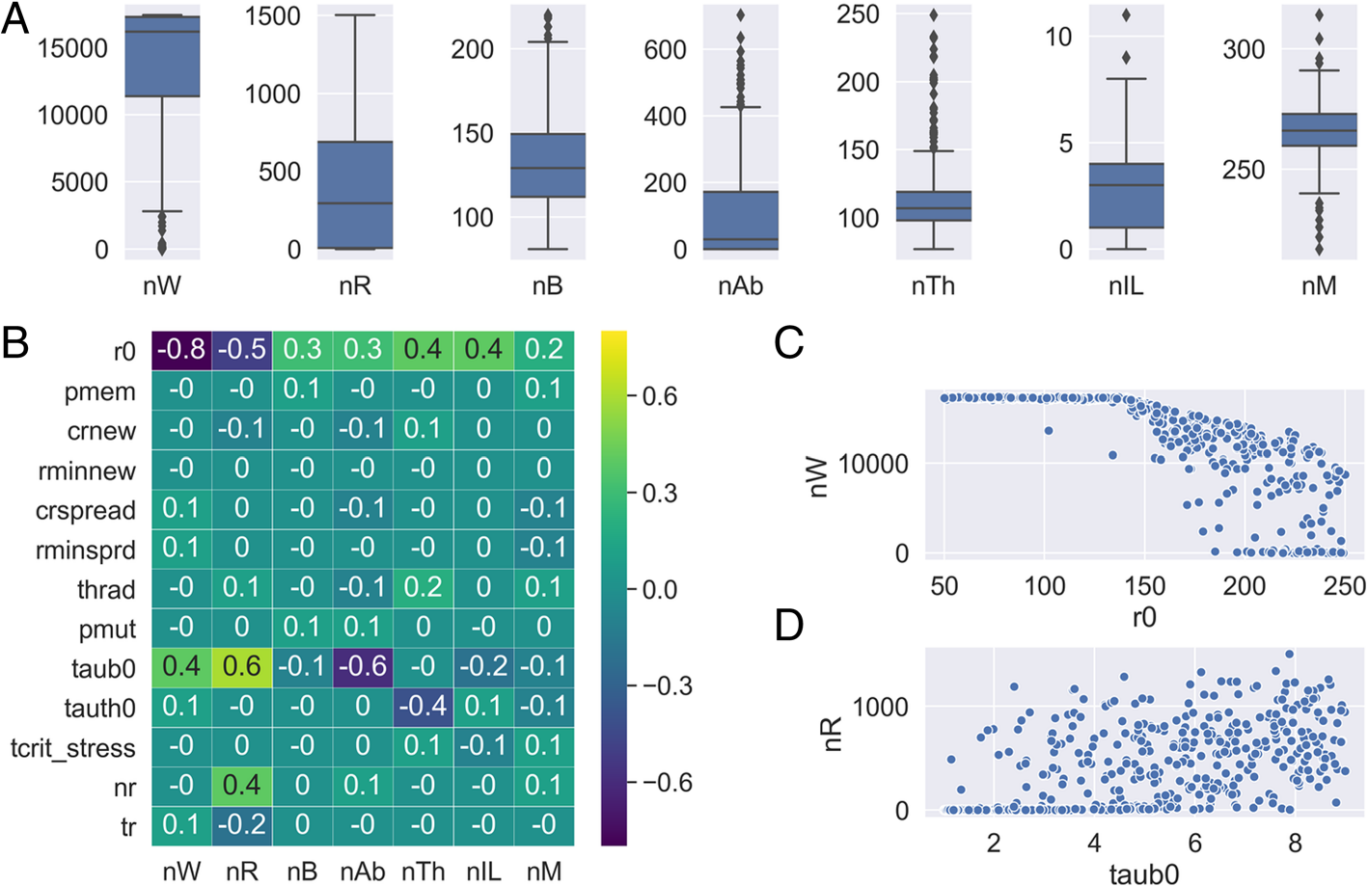
Sensitivity analysis to investigate how 13 critical input parameters impact certain output values 5 days after a random type of infection. **a** Value distribution of the input parameters **b** Pearson correlation coefficient between input parameters (horizontal axis) and output variables (vertical axis). Values are rounded to one decimal. **c** Scatter plot of the input parameter r0 (action radius of naive B cells) and the output variable nW (number of self cells). **d** Scatter plot of the input parameter taub0 (mean waiting time between two actions of a B cell) and the output variable nR (number of foreign cells). Descriptions of the output variables: nW, no. selfs; nR, no. foreign cells; nB, no. B cells; nAb, no. antibodies; nTh, no. T cells; nIL, no. danger signals; nM, no. bone marrow cells. Description of input parameters: see Table 1 in Additional file 1 or the file “indat1” in the Github repository of MiStImm

For this purpose, we have chosen 13 critical parameters that we have sampled randomly (by uniform distribution) around its default value (Table 5). The remaining parameters have been fixed (to the same values as in Fig. 5a and b). We have also varied the random seed of the simulations, as usual. Each simulation was stopped at the 305th day (5 days after an initial infection) and the output variables were only evaluated at the end of the simulation (see Additional file 2 for the raw data of the 500 simulations). Our main observations are summarized in Fig. 6, which shows the distributions of the output values Fig. 6a, and the correlations between input values and output values Fig. 6b, c and d. Interestingly, most of the input parameters do not correlate with any of the other output variables. We can suppose that this trend would remain if we would analyze all of the input parameters. So, actually, there may be much more unimportant (or even unnecessary) input parameters.

**Table 5:** Input parameters for sensitivity analysis

Parameter	Type	Default	Min	Max
r0	int	150	50	250
pmem	float	0.3	0.1	0.5
crnew	float	0.9	0.1	1.7
rminnew	int	5	1	9
crspread	float	0.9	0.1	1.7
rminsprd	int	5	1	10
thrad	int	80	50	250
pmut	float	0.4	0.1	0.7
taub0	float	5	1	9
tauth0	float	2	1	9
tcrit_stress	float	2	1	3
nr	int	350	200	500
tr	float	60	40	80

Values were sampled randomly with uniform distribution around its default value (between Min and Max). Borders of the ranges were chosen rather intuitively, appropriate to the meaning of the parameters, with the rule that the default value should be in the middle of the range

Another interesting observation is that the largest correlation is between the input parameter r0 (action radius of naive B cells) and nW (number of self cells). This may be due to the main characteristic of our proposed model (ERS model) that enables a constant weak connection between B cells and self cells. As we increase the radius of naive B cells there will be more reachable self peptides for naive B cells and it turns into a catastrophe, actually, an autoimmune reaction. The conclusion is that if we want to simulate a healthy (normal) immune reaction we must not set r0 to a value larger than 150 (see Fig. 6c). The second largest correlation was observed between the input parameter taub0 (mean waiting time between two actions of a B cell) and the output value nR (number of foreign cells). This means, not surprisingly, that the more the B cells action, the more efficient the immune reaction is.

## Discussion

When we built our simulator tool MiStImm, we have used some principles that were tested by other researchers in their work. The earliest related computer simulation model is the cellular automaton model Celada and Seiden [8], which is focused, similarly as MiStImm, on the processes that are important for the initiation and regulation of the humoral immune response. All entities (A cells, B cells, T cells, antigens, antibodies) of the Celada-Seiden model along their structural elements appear in MiStImm (except the A-cells), however, we used a more complex model of affinity maturation. Another closely related model is an agent-based model, the C-IMMSIM [19, 20]. The main differences between C-ImmSim and MiStimm are that C-Immsim represents pathogens and lymphocytes receptors by their amino acid sequences and use bioinformatics methods for T and B cell epitope prediction. The Basic Immune Simulator (BIS) [18] is also a closely related agent-based computing model that is intended to study the interactions between innate and adaptive immunity and demonstrated that the initial innate response is crucial for an appropriate adaptive response. [21] developed SIMISYS, which is also a cellular automata model of the human immune system. It uses tens of thousands of cells and innate and adaptive components of the immune system. In particular, the model contains macrophages, dendritic cells, neutrophils, natural killer cells, B cells, T helper cells, complement proteins, and pathogenic bacteria. Reference [33] investigates a hypothesis about B cell hypermutation and affinity maturation using both individual particle-based stochastic and concentration-based non-spatial non-stochastic, ordinary differential equation models. A B cell model developed in [34] has partly similar ideas as our B cell model, but differs from MiStImm in the representation of ligands that are encoded by bit strings and their distances are measured by the number of mismatches (Hamming distance) (like in the above-mentioned IMMSIM and C-ImmSim models). Similar (but not identical) to our ERS model is the mathematical model of T cell mediated suppression of [35], where tolerance is also based on ubiquitous and constitutive self-antigens, which select and sustain clones of specific regulatory (R) cells, and which are similar to our Treg cells. In their model, R cell populations represent typically between 30% and 95% of the total T cells in the periphery. It is an important difference to the widely accepted view in which conventional regulatory CD4+CD25+ T cells (Treg) usually make up only about 5–10% of CD4+ T cells [36]. R cells perform their function through linked recognition of the APCs (antigen presenting cells). Also in their model, immune responses to foreign antigens are achieved by displacing the self-antigens from the APCs, leading to a loss of R cells if the foreign antigen introduction entails a sharp increase in the number of foreign antigen carrying APCs.

Predictions of our theoretical model and the outcomes of the in silico experiment of the Results are supported by numerous clinical trial observations. As a result of the cancer immunotherapy revolution hundreds of clinical trials of the newly approved immunotherapies are now under way to improve responses. Not unexpectedly, the 2018 Nobel Prize in Physiology or Medicine was awarded to James P. Allison and Tasuku Honjo for their development of cancer therapy by blockade of co-inhibitory signals. While success stories of terminal cancer patients achieving complete remissions are accumulating, not enough research has been done into the risks of the new therapies. The developers of the inhibitory anti-CTLA-4 antibody started with the premise that a CTLA-4 (cytotoxic T lymphocyte-associated antigen 4) blockade would selectively target anti-tumor T cells [37]. Although the anti-CTLA-4 antibody improved survival in a minority of metastatic melanoma patients, the vast majority suffered autoimmune-related adverse events (irAEs) [38]. While the conventional nonself centered T cell activation models (implemented as the CRS model in MiStImm) are unable to explain the widespread and dose-dependent irAEs, our self-centered T cell activation model (implemented as the ERS model in MiStImm) can [2, 3]. The reason for this that the nonself-centered models eliminate self-reactive immune cells to ensure that an activation signal can exclusively originate from a foreign/mutated antigen. However, there is evidence for that immune cells require cognate receptor engagement with ubiquitous self antigens in their ’flight for survival’ [39]. During such engagements T cells receive ’tonic’ signal one (TCR signal) generated by positively selecting self-peptide/MHC, which promotes activation and homeostatic survival of T cells in the periphery (see regulatory T cells of the ERS model in MiStImm). Furthermore, there is evidence for control of such tonic TCR signals by co-inhibitors [40]. This is consistent with a critical role for co-inhibitors early in life to establish tolerance in the first T cells that seed the periphery ([40–42]. In addition, the ability of TCRs to interact with tonic self-peptide/MHC ligands opens the possibility that co-inhibitor blockade causes T cell effector activity to spill over onto nearby healthy cells. Increased collateral damage is indeed seen during immune responses where a co-inhibitor is lacking [43]. Altogether, the above concepts suggest that all T cells are temporarily activated, expressing co-inhibitors such as CTLA-4 that can then be targeted by anti-CTLA-4 antibodies. This is consistent with the immunological homunculus concept of Irun Cohen, who suggested that the immune system continuously responds to self [44]. Prolonged therapeutic overstimulation of T cells by antibodies that target their negative regulators (immune checkpoint, IC) such as CTLA-4 and the programmed cell death protein 1 pathway (PD-1/PD-L1) led to a breakthrough in the treatment of a variety of malignancies. While three generations of IC immunotherapy have been developed since Ref. [45, 46], the safety of IC blockade is still an unresolved, timely and sensitive issue in the context of advanced cancer patients. By now Science has acknowledged that these patients are “human experiments” of the autoimmune process [47]. Notwithstanding, we could not find a paper (other than our own) that deduced the widespread irAEs based on the similar outcomes of the TGN1412 and ipilimumab trials despite the fact that the number of ipilimumab papers has increased from 144 (in 2011) to 2585 (PubMed search as of October, 2018). As a result of the impaired self-tolerance, irAEs may present with a broad clinical spectrum that mainly involves the gut, skin, endocrine glands, liver, and lung but can potentially affect any tissue, and their incidence may reach up to 90% of patients [6, 7]. Actually, the Nobel committee emphasized that a crucial aspect in the future development of checkpoint inhibitor therapies is to improve understanding of events leading to adverse events.1 Since the use of immunotherapy is becoming more common, and is expected to develop into first- and second-line treatments, immunotoxicity and autoimmunity are emerging as the nemesis of immunotherapy. Based on our self-centered theory, we have addressed the controversy regarding the safety–efficacy issue in immunotherapy trials and argued that the price we pay for reversing immunosuppression in cancer by a prolonged immune checkpoint blockade is the generation of uncontrolled T-cell activation [48–51]. We predicted that harnessing the unleashed autoimmune power of T cells by low dose IC blockade could be rewarding to defeat cancer. Using our prediction, Ref. [52, 53] have developed just such a promising combination therapy, which was safely and successfully administered to heavily pretreated stage IV cancer patients who had exhausted all conventional treatments.

## Conclusions

We described the MiStImm simulation tool that was made to investigate some important characteristics of immune development, starting from conception and ending some time after birth. Results of some computer experiments were discussed. An important part of the latter was the comparison of the CRS and ERS theoretical models. We think that it is likely that evolution preferred adaptive immune systems whose basic mechanism is closer to the ERS model than to a CRS model because ERS gives better results to overcome a critical primary infection. We hope that our ideas and our computational model may encourage investigations about the problems raised in this paper, using both in vitro and in vivo experiments. We would especially like to see experiments clarifying questions about self-nonself discrimination in a primary infection.

## Availability of data and materials

**Project name:** MiStImm

**Project home page:** https://github.com/kerepesi/mistimm

**Operating system(s):** Windows

**Programming language:** C

**Other requirements:** -

**License:** -

**Any restrictions to use by non-academics:** -

## Additional files

Additional file 1 Appendix. Description of the mathematical model of the event sequence (section 1), the logistic function (section 1) Behavioral rules of the components (agents) of the model (section 3-8). Input parameters for the simulation program (section 8). (PDF 241 kb)

Additional file 2 Data of sensitivity analysis. Results of sensitivity analysis of input parameters. (XLSX 57 kb)

## Abbreviations

BCR: B cell receptor
CRS: Conventional role of self model
ERS: Enhanced role of self model
IrAE: Immune-related adverse event
MHC: Major histocompatibility complex
MiStImm: Microscopic stochastic immune simulator
SA: Sensitivity analysis
TCR: T cell receptor
Treg: regulatory T cell
