# Supplementary material for: MiStImm: an agent-based simulation tool to study the self-nonself discrimination of the adaptive immune response
Source: Theor Biol Med Model. 2019 May 2;16:9. doi: 10.1186/s12976-019-0105-5 (PMC6498635; doi:10.1186/s12976-019-0105-5)
Supplement: Supplementary file 1 — Appendix. Description of the mathematical model of the event sequence (section 1), the logistic function (section 1) Behavioral rules of the components (agents) of the model (section 3-8). Input parameters for the simulation program (section 8). (PDF 241 kb) [file 12976_2019_105_MOESM1_ESM.pdf]

SOFTWARE

Appendix of “MiStImm: an agent-based simulation tool to study the self-nonself discrimination of the adaptive immune response”

Csaba Kerepesi[1*], Tibor Bakács[2] and Tamás Szabados[3]

*Correspondence: kerepesi@sztaki.hu
[1]Institute for Computer Science and Control, Hungarian Academy of Sciences, Kende u 13-17, 1111 Budapest, Hungary
Full list of author information is available at the end of the article

Contents

1 Mathematical model of the event sequence 1
2 Logistic function 3
3 Self cells 3
4 Foreign antigens 4
5 Bone marrow cells 4
6 Th cells 4
7 B cells 7
8 Antibodies 12
9 Input parameters for simulation runs 12

1 Mathematical model of the event sequence

Mathematically, the interactions of the components and other events in the simulation are described by a continuous time, finite state, time-homogeneous Markov process, see e.g. [1]. A Markov process is a memoryless stochastic process: if we specify the present state of the system, then we may forget about its history when we want to investigate its behavior in the future. More precisely, if the possible states of the system are denoted by the natural numbers $1, 2, \ldots, M$, and X_t is the random state of the process at time $t \geq 0$, then the process is described by the transition probabilities

$$P_{i,j}(t) = P(X_{t+s} = j \mid X_s = i) \qquad (i, j = 1, \ldots, M; \quad s, t \geq 0).$$

Let $\mathbf{P}(t) = [P_{i,j}(t)]_{i,j=1}^{M}$ and suppose that $\mathbf{P}(0) = \mathbf{I}$ and $\lim_{t\to 0^+} \mathbf{P}(t) = \mathbf{I}$, where $\mathbf{I}$ is the identity matrix. Then it is well-known that

$$\mathbf{P}(t) = e^{\mathbf{Q}t} = \mathbf{I} + \mathbf{Q}t + \frac{1}{2}\mathbf{Q}^2t^2 + \cdots, \qquad \mathbf{Q} = [q_{i,j}]_{i,j=1}^{M},$$

where $\mathbf{Q}$ is the infinitesimal generator of the Markov process. Thus

$$P_{i,j}(t) = \delta_{i,j} + q_{i,j}\,t + o(t) \quad as \quad t \to 0^+, \tag{1}$$

where $\delta_{i,j} = 1$ if $i = j$ and 0 otherwise, and $o(t)/t \to 0$. It means that the probability of a transition from state i to a state $j \neq i$ is determined by the rate $q_{i,j} \geq 0$; $q_{i,i} = -\sum_{j \neq i} q_{i,j}$.

Let us recall that when one has a Markov transition probability

$$P_{i,i+1}(t) = qt + o(t), \quad P_{i,i}(t) = 1 - qt - o(t) \quad as \quad t \to 0^+,$$

then dividing the time interval $[0, t]$ into n equal subintervals, it follows for the corresponding Markov process Y_t when $Y_0 = 0$ that

$$P(Y_t = k) = \lim_{n\to\infty} nk \left(\frac{qt}{n} + o\left(\frac{t}{n}\right)\right)^k \left(1 - \frac{qt}{n} - o\left(\frac{t}{n}\right)\right)^{n-k}$$

$$= e^{-qt}\frac{(qt)^k}{k!} \quad (t \geq 0, k = 0, 1, 2, \ldots).$$

Thus Y_t is a Poisson process, and so the *holding time* $T := \inf\{t \geq 0 : Y_t \neq 0\}$ is *exponentially distributed*:

$$P(T \geq t) = e^{-qt} \qquad (t \geq 0).$$

Hence it follows from (1), that if $X_s = i$, the holding time $T_i := \inf\{t \geq 0 : X_{s+t} \neq i\}$ is also exponentially distributed:

$$P(T_i \geq t) = e^{-Q_i t} \quad (t \geq 0), \quad Q_i := \sum_{j \neq i} q_{i,j} = -q_{i,i}. \tag{2}$$

Thus one can realize the Markov process $(X_t)_{t\geq 0}$ by assigning to any potential random event an independent exponential clock with rate $q_{i,j}$ $(j \neq i)$, supposing that the present state of the system is $X_s = i$. When the first clock rings, say, the jth one, the corresponding event, that is, the change from state i to j, occurs with rate $q_{i,j}$.

The simulation uses the well-known fact that when there are independent exponential clocks with rates $q_{i,j}$ $(j \neq i)$, then the fastest event has also exponential clock with rate $Q_i := \sum_{j \neq i} q_{i,j}$, see (2). So at any step, it is enough to generate a single exponential random number with rate Q_i. Also, the probability that the event j has occurred, is equal to $q_{i,j}/Q_i$ $(j \neq i)$, whose sum is 1. Thus one generates a uniform random number in $[0, 1)$, and its value determines which one of the concurrent events has occurred.

The simulation has finitely many components at any time t: helper T cells, B cells, antibodies, interleukins, self cells, and foreign antigens. Presently, other than helper type T cells or other antigen presenting cells besides B cells are not represented in

our computational model. Each component has a number of characteristics (parameters) and certain attached random simulation events or subprocesses of events that may occur at random. A potential simulation event can be, for example, a division of a cell or an interaction of a component with a randomly chosen partner. The occurrence of such an event may cause several changes in the model, like births, deaths, and updates of parameters. Because of the births and deaths, our mathematical model is somewhat more general than the simple Markov model described above: the size M of the system changes with time in general. However, it does not cause much difference. We set the model parameters so that explosion does not occur and hence the number of potential events remain finite for all t. At any step, one has to establish the number of independent exponential clocks $M(s)$, and determine their actual rates $q_{i,j} = 1/\tau_{i,j}$ $(1 \leq j \leq M(s), j \neq i)$, where $\tau_{i,j}$ is the *mean holding time* of the jth component. Then the simulation starts again with the new settings.

2 Logistic function

In nature it is typical that when the size of a certain cell population gets larger the per capita birth rate in the population decreases. Thus the size of a population first increases fast, later it slows down, and at the end it gets relatively stable. So to control birth rates and other quantities we use a class of logistic functions:

$$g_{\theta,\eta}(x) := \frac{\theta^\eta}{\theta^\eta + x^\eta} = \left(1 + \left(\frac{x}{\theta}\right)^\eta\right)^{-1} \qquad (x \geq 0; \theta > 0, \eta > 0). \tag{3}$$

This formula describes a decreasing function which is equal to 1 for $x = 0$, 1/2 for the threshold value $x = \theta$, and goes to 0 as $x \to \infty$. Its parameters θ and η are set from case to case. We set the model parameters so that explosion does not occur. In fact, the number of components should always remain in the biologically feasible domain.

3 Self cells

At the beginning of the simulation (at time zero), there is a number (default: 3) of different types of non-immune self cells (briefly: *self cells*), each with a given initial population size (default: 150). A certain type of self cells is represented by its position (x_S, y_S) in the antigen lattice and its peptide (x_P, y_P) in the peptide lattice (see Fig.2 in the main paper). Each type of self cells comes with a birth event with a given initial rate (that is, with a given initial average waiting time τ_{s0} between divisions). If the size of the population of a specific self cell at a certain time t is $s = s(t)$, then the conditional expected waiting time between two divisions in this population is

$$\tau_s = \frac{\tau_{s0}}{s\, g_{\theta,\eta}(s)} = \frac{\tau_{s0}}{s}\left(1 + \left(\frac{s}{\theta}\right)^\eta\right), \quad (\eta > 1). \tag{4}$$

Formula (4) indicates that when the number s of a type of self cells becomes significantly larger than its threshold value θ its division rate gets close to zero. For the sake of simplicity, the natural death process of self cells is not represented in

the model, so, more accurately, (4) should be called the effective growth model of self cells. We assume that the concentration of each type of self antigens in the humoral phase is directly proportional to the number of self cells carrying this antigen. Similar method was used for the birth event of every simulation component (agent).

4 Foreign antigens

After birth, different pathogens may enter the body, perhaps several times (e.g. repeated infections with the same pathogen). A foreign antigen is represented by its position (x_F, y_F) in the antigen lattice and its peptide (x_{FP}, y_{FP}) in the peptide lattice. A foreign cell comes with an initial population size and a birth process with a given initial rate (that is, with a given initial average waiting time τ_{f0} between divisions). If the size of the population of a specific pathogen at a certain time t is $f = f(t)$, then the conditional expected waiting time between two divisions in this population is

$$\tau_f = \frac{\tau_{f0}}{f\, g_{\theta,\eta}(f)} = \frac{\tau_{f0}}{f}\left(1 + \left(\frac{f}{\theta}\right)^{\eta}\right). \tag{5}$$

For the sake of simplicity, the natural death process of foreign antigens is not represented in the model. So (5) should be more accurately called the effective growth process of foreign antigens.

5 Bone marrow cells

Specifically, there is a population of *bone marrow cells*, handled separately from other self cells, with a given initial population size. The case of bone marrow cells is special because it comes not only with a birth rate, but, with given rates, bone marrow cells also produce naïve B cells and Th cells. Naïve B and Th cells have randomly determined BCR and TCR shapes that are uniformly distributed on the antigen and peptide lattices, respectively.

6 Th cells

While different types of non-immune self cells and foreign cells (pathogens) are treated as populations, B and Th cells are handled individually in the simulation. Pre-Th cells are born in the bone marrow. The birth of a pre-Th cell initiates its own (natural) death event, a Th cell action process and a Th cell activation control process.

Th cell recognition region Each Th cell has a recognition region in the peptide lattice. If a TCR is described by the point (x_T, y_T), then the corresponding recognition region is a square with center $(x_T, -y_T)$ and radius r_T. The radius of the TCR is a constant, there is no hypermutation or affinity maturation for Th cells. The recognition region describes the potential shapes of antigens with which a TCR can bind: the smaller the distance between a peptide (x_P, y_P) located on an MHCII and the center $(x_T, -y_T)$ of the recognition region of the TCR, the better the fit.

Thymus To each pre-Th cell there is assigned a random simulation event that places it into the thymus. Here the Th cell goes under a negative and a positive selection process. *Negative selection* kills pre-Th cells that are *closer to one of the self-peptides* than a minimum radius r_{min}; negative selection occurs with a given large probability (dafault: $p_N = 0.99$). *Positive selection* kills pre-Th cells that are *farther from each self peptide* than a maximum radius r_{max}; positive selection occurs with a given, relatively smaller probability, typically $p_P = 0.9$. This way, some of the randomly generated Th cells that cannot bound self-peptides may still survive and they can become infection or mutation specific Th cells later. The *degree of maturity* of a naïve Th cell is 0. In ERS model, if a TCR is in the *characteristic ring* around the reflected image of some self-peptide (see Fig.2B in the main paper), that is, $r_{min} < d(z_P, \overline{z_T}) < r_{max}$, then it is called a *regulatory Th cell* and its degree of maturity is set to 2. Here $\overline{z_T} := (x_T, -y_T)$ is the center of the recognition region of the Th cell and $z_P := (x_P, y_P)$ represents the shape of a self-peptide. In ERS model, a regulatory Th cell has double role. On one hand, it takes part in the controlling role of the regulatory T cell repertoire, but it can also act as a Th cell. Other Th cells that have survived the negative and positive selections, but are outside of the characteristic ring of each self-peptide, are called *potential infection or mutation specific Th cells*, and their degree of maturity is set to 1. In CRS model, we turn off the positive selection of T cells that causes large growth of the T cell population, so simultaneously we need to decrease the expectation of the waiting time between two births of T helper cells in the bone marrow (tauthm $= 5 \to 30$) to ensure that the average number of Th cells be the same as in the case of ERS model. So the number of potential infection specific Th cells are larger in the CRS model than in the ERS model.

Th cell actions In both the ERS and CRS model, for each Th cell, there is a sequence of actions, with exponential random waiting times between two actions. At each action the Th cell is to randomly choose one of the potential target MHCII+peptide complexes in its recognition region. The closer an MHCII+peptide complex to the center of the recognition region, the bigger its chance of being selected.

Th cell activation control process In the ERS model, it is a sequence of frequently occurring random events whose purpose is to check and possibly change the *state of activation* of a Th cell. A Th cell can be in a state of *activated* or *non-activated*. This process checks if this Th cell has received danger signal in a critical period of time before this check. If the result of this check is "yes", then the Th cell is set to "activated" (stress=1); otherwise it is set to "non-activated" (stress=0). An "activated" Th cell starts an interleukin secreting process. This process is a signal of its activated state for "activated" B cells in its environment. We use the symbolic names "interleukins" in this paper, without specifying the exact type of these interleukins. An "activated" Th cell begins *cell division of intermediate kind*. Division of the intermediate kind is different from the weak or strong kind. In the CRS model we use only division of the strong kind.

Self-nonself discrimination It is important that in the ERS model, self-nonself discrimination is solved by the *complete repertoire of Threg cells.* When a *regulatory* Th cell (degree of maturity is 2) bounds with intermediate affinity a B cell's MHCII+peptide complex which has state "non-activated", then with high confidence it means that the peptide is a *self-peptide.* This contact initiates a *division of weak kind* for both this regulatory Th cell and the attached B cell. This weak division helps to stabilize this interaction among three partners: self-cells, B cells that can react to self, and regulatory Th cells that can attach to this self-peptide with intermediate affinity. It is important that B cells that can contact Threg cells with all their MHCII-peptide complexes cannot start an intermediate or strong division process. It gives the important *inhibitory effect* of Threg cells. This way, B cells that react to self are in a state of "non-activated" permanently with large probability. When a Th cell that has already went through the thymus, obtains danger signal then it may begin a non-specific *division of intermediate kind* and may start to secrete interleukins to start *division of intermediate kind of activated B cells.* If a Th cell has already went through the thymus, but it is not a regulatory Th cell (thus its degree of maturity is 1), the target is an activated B cell, and the distance of attachment satisfies $d(z_P, \overline{z_T}) < \frac{r_{min}}{2}$, then with high confidence it means that the peptide is *foreign or mutated self.* Here $\overline{z_T} = (x_T, -y_T)$ is the center of the recognition region of the Th cell, $z_P := (x_P, y_P)$ is the point representing the peptide, and r_{min} is the inner radius of the characteristic ring around the reflected image of self-peptides. Remember that because of the negative selection, such short distance between a self-peptide and the center of recognition region is extremely unlikely. Then both this B cell and Th cell are very likely useful tools to fight against an infection. As a result, this interaction may initiate a *division of strong kind* both in the affected B and Th cells, plus stimulates the secretion of danger signal (in the B cell) and interleukins (in the Th cell). Strong division of a B cell implies its hypermutation with given probability as well. This is a *direct help* of the Th cell for the affected B cell.

Th cell divisions The probability of division of a Th cell may depend on several factors. It may get bigger when the distance d between the MHCII+peptide complex and the TCR is smaller (i.e., the complementarity is better). It gets smaller when the number n_0 of all TCR's is large (i.e., the concentration of Th cells is already large). It gets smaller when the number n_1 of TCR's in a neighborhood of the Th cell is large (i.e., the local concentration of Th cells is already large). The formula for the probability of division is given by a somewhat different formula for the strong division; namely, weak and intermediate divisions do not depend on the complementarity distance d. The reason is that weak reaction by definition have relatively uniform distance between Threg cells and a self antigen, see the rings of the ERS model. Also, intermediate reactions are by definition non-specific, with almost arbitrary distance d. When simulating CRS models, we use only strong division. The probability of a *division of weak kind* of a Th cell is given by

$$p_{T,w} = k_w \, g_{\theta_{n0},\eta_{n0}}(n_0) \, g_{\theta_{n1},\eta_{n1}}(n_1). \tag{6}$$

The purpose of division of weak kind is to establish a stable contact between self antigens, B cells reacting to self with a weak affinity, and Threg cells reacting to self peptides with an intermediate, standard affinity. The probability of a *division of intermediate kind* of a Th cell is given by

$$p_{T,m} = k_m \, g_{\theta_{n0},\eta_{n0}}(n_0) \, g_{\theta_{n1},\eta_{n1}}(n_1). \tag{7}$$

The purpose of division of intermediate kind is to create a fast, non-specific immune reaction to a new, typically quickly growing number of nonself antigens. The growing amount of Th cell help (interleukins) can help the division of intermediate kind of B cells that are able to bind the new nonself antigens in the humoral phase. The probability of a *division of strong kind* of a Th cell is given by

$$p_{T,s} = k_s \, g_{\theta_d,\eta_d}(d) \, g_{\theta_{n0},\eta_{n0}}(n_0) \, g_{\theta_{n1},\eta_{n1}}(n_1). \tag{8}$$

The purpose of division of strong kind of Th cells is to initiate a strong immune reaction when infection or mutation specific Th cells appear and can bind infection or mutation specific B cells. Important requirements to such a division that the binding distance satisfy $d < \frac{r_{min}}{2}$ and the attached MHCII be "activated". These requirements can guarantee with large probability that this strong reaction is not arising against self. Then this Th cell becomes "strongly activated" (stress=2). This condition is independent of danger signal. For simplicity, the constants k_w, k_m, k_s above are typically set to 1.

Regulatory Th cells As we saw above, the regulatory Th cell repertoire plays a most important controlling role in self–nonself discrimination in the ERS model. When simulating CRS models we do not use Thregs explicitly, since their conventional role is to prevent autoimmunity, and when we compare the ERS and CRS models, autoimmunity is avoided. Starting in the fetus, and throughout the entire life span, they give a faithful mirror-image of the self-peptide repertoire.

- They regularly visit B cells having only self-peptides on their MHCII and inhibit their strong division, but support their weak division.
- They are players in normal Th cell roles, like helping non-specific intermediate type and specific strong type division of B cells. They can also secrete interleukins.

7 B cells

Naïve B cells are born in the bone marrow. The birth of a B cell initiates its own (natural) death event, B cell action process, and B cell activation control process, each with separate rate. Each B cell carries a number (default: 3) of MHCII molecules.

B cell recognition region Each B cell has a recognition region in the antigen lattice. If a BCR is described by the point (x_B, y_B), then the corresponding recognition region is a square with center $(x_B, -y_B)$ and radius r_B (see Fig.2 in the main paper). The radius of the BCR of a naïve B cell is a given constant, while B cells that are

born in the periphery after hypermutation may have smaller radii. The BCR $z'_B = (x'_B, y'_B)$ of a hypermutated B cell offspring is determined at random, uniformly on a square around the mother BCR. *Thus there is only a chance that its affinity to a given antigen* $z_A = (x_A, y_A)$ *is higher*, that is, the distance $d(z_A, \overline{z'_B})$ is smaller than that of its mother cell. The radius r'_B of a hypermutated offspring will be smaller than that of its mother cell depending on the above distance: $r'_B = c\,r_B + r_0$. Default values are $c = 0.9$ and $r_0 = 5$. This effect may increase the affinity of some "lucky" offspring to the given antigen. In sum, the recognition region describes the potential shapes of antigens with which a BCR can bind: the smaller the distance between an antigen $z_A = (x_A, y_A)$ and the center $\overline{z_B} = (x_B, -y_B)$ of the recognition region, the better the fit between the antigen and the BCR.

B cell action process For each B cell, there is a sequence of actions, with independent exponential waiting times between two actions. At each action the B cell is to randomly choose one of the potential target antigens in its recognition region. A target can be another B cell, an antibody, a non-immune self cell, or a foreign antigen. The closer an antigen $z_A = (x_A, y_A)$ to the center $\overline{z_B} = (x_B, -y_B)$ of the recognition region, the bigger its chance of being selected as the next target. The chosen target can be killed only if the above distance is smaller than the recognition radius r_B of the B cell, that is, $d(z_A, \overline{z_B}) < r_B$. The smaller this distance, the larger the probability that the antigen will really be destroyed. Since smaller distance represents stronger affinity in the model, it means longer attachment between an antigen and the BCR. So this condition is equivalent to the fact that a target can be killed if it is bound to the BCR for a long enough time. When the chosen target is destroyed, its peptide is placed on one of the MHCII's of the B cell. The MHCII selected is primarily an empty one; when all of the MHCII's are already loaded, then one of them is chosen at random to replace the old peptide by the new one.

B cell negative selection filter in the bone marrow To each naïve (immature) B cell there is assigned a random event that places it into a negative selection filter in the bone marrow. Negative selection kills B cells that are *closer to one of the self-antigens* than a minimum radius r_{minb}; negative selection occurs with a given large probability (default: $p_{Nb} = 0.99$). The *degree of maturity* of a naïve B cell is 0. A B cell that has survived the negative selection is called a *mature B cell*, and their degree of maturity is set to 1. Only B cells with degree of maturity ≥ 1 can function as normal B cells.

B cell activation control process In the ERS model, it is a sequence of frequently occurring events whose purpose is to check and possibly change the *state of activation* of a B cell. It is not used in the CRS models. The main parameter is the *critical time* t_{crit}. Each of the MHCII carried by a B cell can be in a state of *"activated"* or *"non-activated"*. An empty MHCII is not "activated" by definition.

- A given non-empty MHCII is set to "non-activated" when the time elapsed since the last event effecting this MHCII is less than t_{crit}. Such an event can be a regulatory Th cell attaching to this MHCII, or placing a new peptide on this MHCII.

- A given MHCII is set to "activated" when the time elapsed since the last event effecting this MHCII is greater than or equal to t_{crit}.

Similarly, a B cell can also be in a state of *"activated"* or *"non-activated"*.

- When its each MHCII is in the state of "non-activated", the B cell itself is set to state of "non-activated".
- When at least one of its MHCII is "activated", then the B cell is set to "activated".

An "activated" B cell starts an *danger signal* sending process. This process is a signal of its activated state for Th cells in its environment. An "activated" B cell may start a *cell division of intermediate kind* if it obtains help from non-specific Th cells. Help may come as interleukins produced by Th cells, that has arrived in a critical period of time before this check. (This kind of cell division cannot occur with plasma cells or memory cells.) Division of the intermediate kind is different from the weak or strong kind. Here the activation (stress) level is 1. In the case of *cell division of the strong kind*, which occurs by the help of infection or mutation specific Th cells, the activation (stress) level is 2.

B cell division and maturity Each B cell has *a degree of maturity.* A naïve, immature B cell has degree 0, while B cells that have survived a negative selection filter in the bone marrow are mature B cells, having degree of maturity 1 first. Mature B cells may encounter antigens at the periphery. A B cell division can be the result of an encounter with an antigen which is escorted by a direct or indirect (via interleukin) help from a Th cell. At each division of a B cell, one of the two offspring inherits all characteristics of the mother cell (let us call it the *first offspring* for explicitness), while the other offspring (let us call it the *second offspring*) may undergo hypermutation with given probability. The first offspring inherits the mother's MHCII-peptide complexes, while the second offspring starts with MHCII molecules with a default (non-specific) peptide. The second offspring after the first division has a degree of maturity 2. The result of a hypermutation is a B cell with randomly shaped BCR. The possible shapes are uniformly distributed on a square of the antigen lattice, with given radius around the mother BCR.

A second division may lead to two different outcomes with given probabilities: the second offspring can be either a *memory cell* (degree=3) or a *plasma cell* (degree=4). A memory cell has the same characteristics as a normal B cell except that its average lifespan is significantly longer (e.g 10 days instead of the standard 3 days). A plasma cell constantly – at random time instants – produces antibodies of the type of its own BCR.

Possibility of division of a B cell arises after contacting an antigen or obtaining Th help in the form of interleukins. The probability of division of a B cell depends on several factors. It gets bigger when the distance d between the antigen and the BCR is smaller (i.e., the complementarity is better), or when the radius r of the recognition region of the BCR is smaller (i.e., the affinity of the B cell is bigger). It gets smaller when the number n_0 of other BCRs in a rectangle around the BCR is small (i.e., the concentration of B cells is already large). Finally, one or two factors can depend on the concentration difference c between the number of targets in the recognition region of the B cell and the number of targets in the reflected image

of the recognition region. If the concentration difference is too small, the B cell may get insensitive. If the concentration difference is too large, the B cell may get anergic.

In the ERS model, the specific formulas for the probability of division in the respective cases of weak, intermediate, and strong B cell divisions are as follows. In the CRS models only the strong division is used. The probability of a *division of weak kind* of a B cell is given by

$$p_{B,w} = k_w\, g_{r_{mb}+\theta_d,\eta_d}(d)(1-g_{r_{mb}-\theta_d,\eta_d}(d))\, g_{\theta_r,\eta_r}(r)\, g_{\theta_n,\eta_n}(n_0)\,(1-g_{n_m,\eta_c}(c)). \quad (9)$$

The purpose of division of weak kind is to establish a stable contact between self antigens, B cells reacting to self with a weak affinity, and Threg cells reacting to self peptides with an intermediate, standard affinity. The first, constant factor k_w is typically 1. The purpose of the second and third factors depending on d is to help those B cells that are at a standard distance from their targets, in the present case, self antigens. The last factor, depending on c intends to guarantee that a large number of antigens, typical for self antigens, be in the recognition region of the weakly dividing B cells. The first parameter n_m here is the actual number of bone marrow cells, which is a common measure of the size of non-immune self cell populations. The probability of a *division of intermediate kind* of a B cell is given by

$$p_{B,m} = k_m\, g_{\theta_d,\eta_d}(d)\, g_{\theta_r,\eta_r}(r)\, g_{\theta_n,\eta_n}(n_0)\, g_{\theta_{c2},\eta_c}(c)\,(1-g_{\theta_{c1},\eta_c}(c)). \quad (10)$$

The purpose of division of intermediate kind is to create a fast, non-specific immune reaction to a new, typically quickly growing number of nonself antigens. The growing amount of B cells that are able to bind the new nonself antigens in the humoral phase even when there exist no infection or mutation specific B or Th cells can give an early start to an effective immune reaction. Activated B cells can release danger signal to initiate a non-specific Th help as well. The value of the constant multiplier k_m is typically 100 to create a fast answer to a new, quickly dividing infection. The probability of a *division of strong kind* of a B cell is given by

$$p_{B,s} = k_s\, g_{\theta_d,\eta_d}(d)\, g_{\theta_r,\eta_r}(r)\, g_{\theta_n,\eta_n}(n_0)\, g_{\theta_{c2},\eta_c}(c)\,(1-g_{\theta_{c1},\eta_c}(c)). \quad (11)$$

The purpose of division of strong kind of B cells is to initiate a strong immune reaction when infection or mutation specific Th cells appear and can bind infection or mutation specific B cells. Important requirements to such a division that an "activated" Th cell binds an "activated" MHCII of this B cell and the binding distance between the reflected image of the TCR and the peptide is smaller than $\frac{r_{min}}{2}$. These requirements can guarantee with large probability that this strong reaction is not arising against self. The value of the constant k_s is typically 200 to create a strong reaction when – tipically – the number of B cells specific to a new infection is very low.

B cell affinity maturation and network memory Like in natural selection, there exists neither intelligent control which would direct genetic mutations toward better fit, nor memory that would save cells from genetically searching a proved wrong "direction". The major effect which has physiological consequences on a B cell is the strength of antigen binding. This is like finding the source of heat in a dark room, using a single thermometer, with no direct sensing of direction and with no memory. The technique the present model applies is a microscopic analog of evolution: hypermutation and selection, with survival of the fittest. Namely, the program uses a stochastic search for best fit (or a stochastic learning process):

- An offspring may be randomly hypermutated, so a random variation is created in the affinity to the given antigen.
- The stronger a B cell can bind a given antigen, the more offspring it can produce.
- When the concentration of the given antigen is decreasing, a competition arises among B cells for the antigen, and those having higher affinity would win in this selection process.

An affinity maturation model has to handle the danger of autoimmunity. Even if naïve Th cells which can strongly bind self peptides are deleted as a result of negative selection in the thymus, and also naïve B cells which can strongly bind self antigens are deleted as a result of negative selection in the bone marrow, still there is the danger that autoimmune B cell clones may be produced as a result of hypermutation. In the presented model there is a double defense against this danger.

- The absence of T cell help in the case of B cells that react strongly to non-immune self antigens inhibits their division. This is an essential difference between self and nonself in the model.
- Since nonself antigens which can start somatic hypermutation typically appear after birth, when the number of self cells is already very large, one can argue that at that time randomly produced self-reactive B cell clones are confronted with an overwhelming quantity of self antigens. As a result, these B cell clones would become anergic [2]. In the model this is simulated in the B cell division process: divisions of a B cell, see (10) and (11), become less frequent when the number of objects in its recognition region becomes overwhelmingly large. The reproduction process of B cells is fastest when the concentration of the complementary antigens is neither too small, nor too large. This is common for both self or nonself antigens in the model, so when nonself overgrows an upper threshold, the model immune system remains practically defenseless against it as well.

As a result of the double defense described above, there will be "holes" in the adaptive immune system, both in the T cell and B cell populations, around the mirror image of non-immune self cells [3, 4]. The negative selection in the model is especially important during early ontogenesis when the smaller population of host cells is vulnerable to self-reactive immune cells. As the individual reaches adult size, the large number of host cells plus the absence of T cell help can alone inhibit reproduction and affinity maturation of immune cells. Then negative selection in the model (like in reality in the thymus) becomes less essential. It is reasonable to expect that after a somatic hypermutation — affinity maturation process the resulted

specific B cell clones may survive for a certain period of time as a local memory. In the model, expansion of certain B cell clones (e.g. as a result of an infection by a foreign antigen), under favorable conditions, stimulates the reproduction of secondary B cells which are complementary to the expanded primary B cell clones and whose receptors are, therefore, similar to the infecting antigens. (Of course, similarity here means a mimicry of a binding partner and not similarity at the molecular level.) Thus a mirroring process ("ping-pong") and a local network memory may develop and last for a longer time, even in the absence of the stimulating antigen. While this memory lasts, repeated infection of the same pathogen is eliminated more efficiently. This network model of immune memory essentially conforms to Jerne's immune network concept [5]. Beside other factors, like longer living memory cells or antigen preserving follicular dendritic cells, this could be a possible explanation of immune memory.

8 Antibodies

A plasma cell is a special kind of B cells, a result of a B cell maturity process. A plasma cell has neither a B cell action event, nor a B cell activation control event. On the other hand, it has an antibody birth and an antibody death process. An antibody has the same shape in the antigen lattice as the BCR of its mother plasma cell.

Antibodies have similar action processes as B cells, but, naturally, when tagging a target, peptide of the target does not appear on an MHCII. The complement sub-system of the immune system is currently not represented in the model, so it is supposed that when an antigen is tagged by an antibody, it leads to the destruction of the targeted antigen with a certain probability.

9 Input parameters for simulation runs

MiStImm can be initialized by approximately one hundred input parameters (Table 1) The parameters can be used to set various immune system models, including the above mentioned ERS and CRS models. Once an immune system model is fixed, further individual settings are available (for example, foreign cell injections with different numbers or types). Different initial random numbers can be set to run different random realizations with the same parameter settings.

Author details
[1]Institute for Computer Science and Control, Hungarian Academy of Sciences, Kende u 13-17, 1111 Budapest, Hungary. [2]Alfréd Rényi Institute of Mathematics, Hungarian Academy of Sciences, Reáltanoda u 13-15, 1053 Budapest, Hungary. [3]Department of Stochastics, Budapest University of Technology and Economics, Müegyetem rkp 3, 1521 Budapest, Hungary.

References

1. Lamperti, J.: Stochastic Processes: a Survey of the Mathematical Theory vol. 23. Springer, ??? (2012)
2. Goodnow, C.C., Crosbie, T., Jorgensen, H., Brink, R.A., Basten, A.: Induction of self tolerance in mature peripheral B lymphocytes. Nature **342**, 385–391 (1989)
3. Goodnow, C.C., Crosbie, J., Adelstein, S., Lavoie, T.B., Smith-Gill, S.J., Brink, R.A., Pritchard-Briscoe, H., Wotherspoon, J.S., Loblay, R.H., Raphael, K., Trent, R.J., Basten, A.: Altered immunoglobulin expression and functional silencing of self-reactive B lymphocytes in transgenic mice. Nature **334**, 676–682 (1988)
4. Goodnow, C.C., Adelstein, A., Basten, A.: The need for central and peripherial tolerance in the B cell repertoire. Science **248**, 1373–1379 (1990)
5. Jerne, N.K.: Towards a network theory of the immune system. Annales d'Immunologie **125**(1-2), 373–389 (1974)

Table 1 The most important parameters of MiStImm. The unit of time is one-tenth of a day.

Parameter	Description	Typical value
comptype	computational model	0: ERS, 1: CRS
medrepr	intermediate interaction	0: off, 1: on
weakrepr	weak interaction	0: off, 1: on
nrmax	the simulation stops at this number of pathogens	5000
nm	initial number of bone marrow cells	5
timmst	starting time of the immune system	100
tmax	the last time instant of the simulation	5000
xmax	size of the antigen lattice	1000
r0	radius of naïve B cells	140
r0s	radius of spreading area of offspring B cells	60
tlifeb	mean life length of a B cell	30
tlifmem	mean life length of a memory B cell	150
pmem	the probability of B cell changing into memory cell	0.3
thrad	radius of Th cells	80
pxmax	the size of the peptide lattice	1000
rminth	threshold radius of negative selection	30
rmaxth	threshold radius of positive selection	50
rminb	threshold radius of negative selection of B cells	140
pmut	the probability of B cell hypermutation at reproduction	0.4
taum	mean time between two divisions of a bone marrow cell	400
taubm	mean time between two births of B cells in b. marrow	30
tauselb	mean time for a B cell to enter negative selection	0.05
taub	mean time between two actions of a B cell	5
taubstress	mean time between two B cell activation checking	0.5
taubil	mean time between the births of IL type 2	0.2
taudil	mean time between the deaths of IL type 2	30
tauab	mean time between two actions of an antibody	0.5
taubab	mean time between two births of antibodies	1
taudab	mean time between two deaths of antibodies	80
tauthm	mean time between two births of Th cells in b. marrow	5
tauthymus	mean time for a Th cell to enter the thymus	0.05
tauth	mean time between two actions of a Th cell	2
tlifeth	mean life length of a Th cell	30
sreprcrit	threshold radius of the strong reproduction	40
dring	radius within which the cc. of Th cells are restricted	10
tcritilb	crit. time between arrivals of two IL type 2 at a B cell	1
tcritth	crit. time between two Th cells arrival at a given MHCII	2
tauprodil1	mean time between two births of IL type 1	0.2
taudil1	mean time between two deaths of IL type 1	30
tauil1	attack rate of an IL type 1	1
tauil	attack rate of an IL type 2	1
tthcrit	crit. time between arrivals of two IL type 1 at a Th cell	1
tauthstress	rate of the Th activation control process	0.5
negselp	threshold probability of Th negative selection	0.99
posselp	threshold probability of Th positive selection	0.9
bselp	threshold probability B cell negative selection	0.99
th*	different theta parameters, see (3)	1–1000
eta*	different eta parameters, see (3)	1–4
kth0, 1, 2	multipliers of Th reproduction, see (6), (7), (8)	1
kb0	multiplier of B weak reproduction, see (9)	1
kb1	multiplier of B intermediate reproduction, see (10)	100
kb2	multiplier of B strong reproduction, see (11)	200
nwtypes	number of types of self cells	1–4
nw	initial number of a specific self cell	150
xw	x-coordinate of a specific self cell	0–1000
yw	y-coordinate of a specific self cell	-500–500
t0w	starting time of a self cell type	0
tauw	mean time between two divisions of a spec. self cell	40
nrtypes	number of types of pathogens	1–4
nr	initial number of a specific pathogen	200–700
xr	x-coordinate of a specific pathogen	0–1000
yr	y-coordinate of a specific pathogen	-500–500
t0r	starting time of a specific pathogen	3000–4000
taur	mean time between two divisions of a spec. pathogen	30–80
